# Critical Roles of Balanced Innate Lymphoid Cell Subsets in Intestinal Homeostasis, Chronic Inflammation, and Cancer

**DOI:** 10.1155/2019/1325181

**Published:** 2019-11-05

**Authors:** Jing Wu, Xinping Lv, Shan Zhu, Tete Li, Hang Cheng, Jingtao Chen

**Affiliations:** ^1^Institute of Translational Medicine, The First Hospital, Jilin University, Changchun 130061, China; ^2^Department of Pediatrics, The First Hospital, Jilin University, Changchun 130021, China

## Abstract

Innate lymphoid cells (ILCs) comprise a recently identified subset of innate immune cells that are mainly localized to mucosa-associated tissues. Although they have not yet been fully characterized, they can generally be divided into ILC1s, ILC2s, and ILC3s. ILCs and their corresponding cytokines act as important mediators of the early stages of the immune response during inflammation, tissue repair, and the maintenance of epithelial integrity. Consequently, the dysregulation of ILC subsets might promote inflammation and cancer. Numerous studies have demonstrated that these cells play an important role in maintaining the microecological balance of the small intestine; however, their specific roles in mediating inflammation in this tissue and tumorigenesis remain unclear and controversial. In this review, we focus on recent progress that has helped to gain a better understanding of the role of ILCs in intestinal homeostasis, chronic inflammation, and cancer. Further focused research on the regulation and role of ILCs in intestinal homeostasis and pathology will help to reveal valuable diagnostic and therapeutic targets for the treatment of intestinal diseases.

## 1. Introduction

Intestinal epithelial cells (IECs) cover the luminal surface of both the small and large intestines of the gastrointestinal tract. As part of the intestinal mucosa layer, IECs are single-layer, columnar cells organized with tight junctions that form a contiguous and relative impermeable membrane [1]. The primary functions of these cells are to absorb water, electrolytes, and dietary nutrients into the body, while restricting the entry of harmful pathogens. IECs not only provide an important physical barrier to microorganisms but also express cytokines and chemokines that interact with mucosal immune cells to maintain immune homeostasis [2, 3].

Innate lymphoid cells (ILCs) are recently identified mucosal immune cells considered the gatekeeper of mucosa-associated tissues such as the gut. Their function is regulated by IEC-secreted cytokines in response to physiological and pathological processes including immune defense, tissue remodeling, inflammation, and cancer [2, 4, 5]. ILCs develop from precursors that express integrin *α*_4_*β*_7_ in the bone marrow, which interacts with the chemokine receptor CXCR6 and adhesion molecule MadCAM-1 when cells migrate to the intestine [6, 7]. ILCs are specifically localized to the lamina propria of the small and large intestines and are rarely replenished from the bone marrow; under both steady-state and homeostasis-disruptive conditions, these tissue-resident cells remain in the intestine and exhibit local self-renewal via the proliferation of tissue-resident progenitor cells [8]. Unlike classical lymphocytes such as T and B cells, ILCs lack antigen-specific receptors. They rapidly respond to environmental challenges and provide immunity to fight against the invasion of a variety of infectious pathogens, all while playing an important role in organ homeostasis by producing factors that act on epithelial cells [9].

Based on their phenotypic and functional characteristics, ILCs can be generally divided into cytolytic and noncytolytic ILCs [4, 10]. Cytolytic ILCs, also referred to as conventional natural killer (NK) cells, release cytolytic effector molecules including perforin and granzyme B, which can kill tumors or virus-infected tissue. In contrast to NK cells, noncytolytic or “helper” ILC populations can be classified into three groups (groups 1–3) [11–13] (Table 1). Group 1 ILCs (ILC1s) are characterized by the production of interferon- (IFN-) *γ* and lack the production of T helper 2- (Th2-) and Th17-associated cytokines. ILC1s express high levels of the transcription factor T-bet and low levels of the transcription factor retinoid-related orphan receptor *γ*t (ROR*γ*t) but lack expression of CD117 [14]. Group 2 ILCs (ILC2s) are characterized by the production of the Th2-associated cytokines interleukin- (IL-) 5 and IL-13 after stimulation by thymic stromal lymphopoietin (TSLP) [15], IL-33, and IL-25 [16]. The development of ILC2s requires the presence of IL-7 [17] and the transcription factors GATA-3 in humans, and the transcription factors GATA-3 and ROR*α* in mice [12, 18, 19]. Group 3 ILCs (ILC3s) are similar to ILC2s with regard to their dependence on IL-7, but they also require the transcription factor ROR*γ*t for their development and function and produce IL-17A and/or IL-22 [12, 20]. This group includes lymphoid tissue inducer cells, which are natural cytotoxicity receptor (NCR)^+/−^ ILC3s.

In addition, recent studies have revealed a regulatory subpopulation of ILCs (called ILCregs) and memory ILCs, which include circulating memory NK cells and tissue-resident memory ILCs [21–23]. ILCregs exist in the gut and control intestinal inflammation via the secretion of IL-10 [21] (Table 1). Tissue-resident memory ILCs exist in the liver or lung and have adaptive features, including virus and hapten-induced memory NK cells, hapten-induced memory ILC1s, and cytokine-induced memory ILC2s [22, 23]. Accumulating evidence also suggests that tissue-resident memory ILCs might represent the innate counterparts of resident memory T (T_RM_) cells owing to some common features [22].

ILCs display certain levels of functional diversity and plasticity. Under the influence of IL-12, IL-18, and IL-1*β*, ILC2s and ILC3s can transdifferentiate into ILC1s, which can in turn transdifferentiate back into ILC2s and ILC3s in the presence of IL-4 and IL-23, respectively [24–27]. Thus, ILCs are important regulators of epithelial barriers, are involved in immune defense, and participate in various diseases of the intestine including inflammation and cancer. Accordingly, gaining a better understanding of the complex biological mechanisms underlying these roles will facilitate the development and recognition of the diagnostic and therapeutic potential of ILCs for the treatment of various diseases. To promote this goal, here, we review the research progress on the physiological and pathological roles of ILCs in immune defense and the maintenance of the intestinal microecological balance to highlight targets for the treatment of intestinal diseases including chronic inflammation and cancer.

## 2. ILCs in Normal Intestinal Tissue

The intestinal epithelium is the largest barrier that isolates an organ system from the immediate environment. ILC subsets have been found to play a role in gut homeostasis in both humans and mice [8, 28]. In healthy conditions, very few ILCs are detected, with NCR^+^ ILCs accounting for 5% and 2% of total lymphocytes in the human and mouse small intestine, respectively [29], and ILC2s comprising approximately 5% of all small intestinal lymphoid cells [30]. Given their localization, ILCs are among the first immune cells to react to invading pathogens and are also involved in maintaining the integrity of the epithelial barrier. Thus, these cells play vital roles in the reciprocal interactions between the gut microbiota and immune system.

Cytolytic ILCs (NK cells) and noncytolytic ILC1s are known as T-bet^+^ and IFN-*γ*-producing subsets, respectively. However, although they share many characteristics, based on recent studies, NK cells and ILC1s develop from different bone marrow progenitors, and therefore, they are substantially different in their tissue tropism, migratory capacity, and effector functions [31, 32]. ILC2s can mediate protection against helminth infection in murine models [33]. In the steady state, most intestinal ILC3s express ROR*γ*t and NKp44 in humans and NKp46 in mice [34]. NCR^+^ ILC3s produce IL-22 upon interaction with the transcription factor aryl hydrocarbon receptor ligand, which can be derived from the diet and microflora [35]. Although ILCs are rarely present in healthy conditions, understanding their characteristics in the steady state will help to further elucidate their possible roles in disease and their potential as targets to prevent or treat these diseases.

## 3. Roles of ILCs in Intestinal Tissue Immune Defense and Maintenance of the Intestinal Microecological Balance

**Figure 1:**
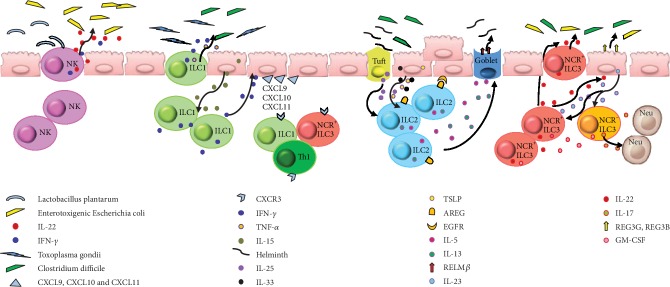
The multiple roles of innate lymphoid cells (ILCs) in intestinal tissue immune defense and tissue remodeling. NK cells can attenuate intestinal damage by producing IL-22 and IFN-*γ* after infection. ILC1s mediate protection via IFN-*γ* and TNF-*α* production after infection and attract CXCR3^+^ leukocyte accumulation. ILC2s produce multiple important effector molecules after activation, promote intestinal repair, and limit parasitic infections. In response to IL-23 stimulation, NCR^+^ ILC3s and NCR*^−^* ILC3s mainly produce IL-22 and IL-17, respectively, and GM-CSF from both kinds of cells, and participate in homeostasis of the intestine.

The functional activity of ILCs requires exposure to the gut microbiota, as shown by a mouse model study in which germ-free or antibiotic-treated mice displayed impaired NK cell activity [36]. Intestinal NK cells interact with many strains of probiotics to maintain the integrity of the epithelial cell barrier, such as interactions with *Lactobacillus plantarum* to attenuate enterotoxigenic *Escherichia coli*- (ETEC-) induced epithelial damage. Moreover, defense against ETEC is considered to involve the stimulation of NK cells to enhance IL-22 production [37] (Figure 1).

The absence of ILC1s in T-bet*^−/−^* mice is linked to their increased susceptibility to enteric infections [38]. ILC1s mediate protective responses during *Toxoplasma gondii* and intestinal *Clostridium difficile* infections via T-bet, with the consequent production of IFN-*γ* and tumor necrosis factor- (TNF-) *α* [6, 39]. Similarly, tissue-resident ILC1s play an essential role in viral infections, enabling the rapid production of IFN-*γ* to limit the early viral burden [40]. IL-15 produced from IECs induces the release of IFN-*γ* by ILC1s, which enhances the expression of chemokines CXCL9, CXCL10, and CXCL11 in IECs, which recruit CXCR3^+^ leukocytes including Th1, ILC1s, and NKp46^+^ ILC3 cells [5] (Figure 1). Furthermore, the transfer of ILC precursors into a lymphoid mouse model promotes the recruitment of monocytes, which helps to limit extensive inflammation [6].

ILC2s express the signature transcription factor GATA-3, as well as CD90, CD127, CD25, IL-25R, and IL-33R, and are distributed throughout the intestinal lamina propria [41, 42]. ILC2s are activated by epithelial cell-derived alarmins such as IL-25, IL-33, and TSLP [43, 44] and produce multiple important effector molecules including amphiregulin (AREG) [45], IL-5, IL-9, and IL-13 [46]. AREG is a ligand of a widely expressed transmembrane tyrosine kinase epidermal growth factor receptor (EGFR) [47], and binding between AREG and EGFR stimulates the proliferation of epithelial cells [48]. An analogous IL-33–ILC2–AREG pathway also plays an important role in intestinal epithelial cell renewal and intestinal repair [45].

During *C. difficile* infection, ILC1s provide immune protection, whereas ILC2s are activated by IL-33 as an essential pathway for in recovery from *C. difficile* infection-associated colitis [49]. Importantly, ILC2s coordinate the inflammatory response to helminth infection in the gut. Stimulation with TSLP, IL-25, and IL-33 induces ILC2s to release cytokines IL-5 and IL-13, which promote mucus and antimicrobial peptide (RELM*β*) production by intestinal goblet cells, which help to limit parasitic infections [33, 41, 50, 51]. Recently, tuft cells were identified as the major source of intestinal IL-25 production, which in turn promotes the production of IL-13 by ILC2s [52, 53] (Figure 1).

ILC3s are involved in defense against bacterial and fungal infection, the regulation of commensal bacteria, and the development and repair of lymphoid tissues. In response to IL-23 stimulation, NCR^+^ ILC3s and NCR*^−^* ILC3s mainly produce the Th17- and Th22-associated cytokines IL-17 and IL-22, respectively. IL-22 plays a critical role in intestinal epithelial injury repair after bacterial pathogen invasion [54]. As primary producers of mucosal IL-22, intestinal ILC3s play a crucial role in protecting against gut bacterial infections [28, 55, 56]. In response to IL-22, epithelial cells secrete antimicrobial peptides (REG3G, REG3B), lipocalin, and mucus to reinforce barrier protection in response to microbial damage. Furthermore, ILC3-derived IL-22 helps to contain gut-associated lymphoid tissue-resident commensal bacteria and to protect intestinal stem cells in graft-versus-host disease models [57, 58]. Epithelial cells can also indirectly regulate ILC3s during interactions with commensal bacteria. For example, IECs can produce IL-25 to suppress the production of IL-22 by ILC3s, whereas IL-7 production by IECs stabilizes the transcription factor ROR*γ*t to boost IL-22 production [59, 60]. Expression of the IL-22 receptor subunit IL-22R*α*1 in intestinal stem cells [61, 62] mediates epithelial regeneration, and constant IL-22 production is essential to maintain barrier integrity and commensal bacteria in a steady state. IL-17A participates in the recruitment of neutrophils, as important effector cells for extracellular pathogen immunity, and induces IECs to express high levels of CXCL1 and CXCL2 [5]. Granulocyte-macrophage colony-stimulating factor (GM-CSF) is another important cytokine produced by both NCR^+^ ILC3s and NCR*^−^* ILC3s, which helps to maintain the homeostasis of mononuclear phagocytes in the intestine [5] (Figure 1). Collectively, these studies demonstrate the importance of the microflora in shaping the development and function of ILCs via direct or indirect interactions with IECs during intestinal tissue immune defense, while maintaining the intestinal micro-ecological balance.

## 4. ILCs in Intestinal Chronic Inflammation

**Figure 2:**
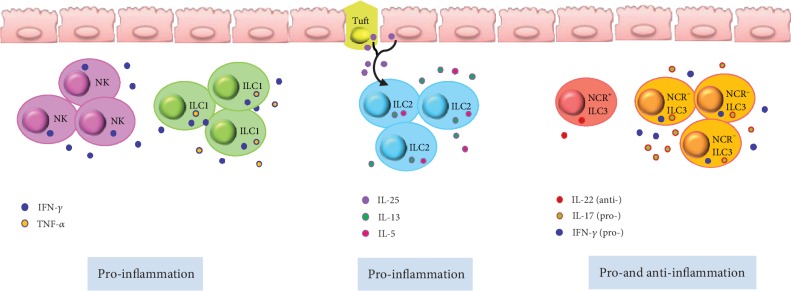
Potential pro- and anti-inflammatory roles of ILCs in intestinal chronic inflammation. NK cells and ILC1s are increased in the colonic lamina propria tissue of inflammatory bowel disease (IBD) patients. NK cells produce IFN-*γ*, whereas ILC1s produce IFN-*γ* and TNF-*α*. ILC2s play a pathogenic role by promoting IL-13- and IL-5-driven inflammation in a mouse model of colitis, which is ameliorated by blocking IL-25. IL22^+^ NCR^+^ ILC3s are decreased dramatically and NCR*^−^* ILC3s are increased under chronic inflammation conditions. The NCR*^−^* ILC3 subset can contribute to intestinal chronic inflammation through the production of IL-17A and IFN-*γ*.

NK cells are involved in the pathogenesis of inflammatory bowel disease (IBD) including ulcerative colitis (UC) and Crohn's disease (CD) [63–65]. Steel's group reported that the number of CD16^+^ NK cells is increased in the colonic lamina propria tissue of patients with IBD [63] and Takayama's group showed that NKp46^+^ NK cells might mediate the pathogenesis of CD via IFN-*γ* production [64] (Figure 2). Ng's group further identified that a subset of CD56^+^ HLA-DR^+^ NK cells in the colonic lamina propria is associated with intestinal inflammation in UC patients [66]. Further, Yusung's group showed that the number of NK cells in the colon decreases after therapy with the immunosuppressive drug 6-mercaptopurine in CD patients [67]. Together, these findings support the contention that NK cells are a very attractive immunotherapy target for IBD patients.

As described previously herein, ILC1s play a critical role in host defense against intracellular pathogens by secreting TNF-*α* and IFN-*γ* in the gut during steady-state conditions. However, under inflamed conditions, the frequency of ILC1s increases resulting in excessive cytokine production. Several groups reported that the frequency of ILC1s is increased in the intestines of patients with CD [14, 68, 69] (Figure 2). Li's group reported that an increase in IFN-*γ*-producing-CD127^+^ ILC1s in the inflamed intestine is associated with disease severity [70, 71], and Bernink's group further reported that under the control of the cytokines IL-12 and IL-23, NKp44^+^ ILC3s can convert into IFN-*γ*-producing ILC1s [14].

ILC2s play a pathogenic role by promoting IL-13-driven inflammation in an oxazolone-induced mouse model of colitis, which could be ameliorated by blocking IL-25 [72] (Figure 2). Monticelli's group revealed that ILC2s mediate tissue protection during intestinal injury by limiting inflammation and promoting epithelial repair through AREG secretion in a mouse model [45]. Bailey's group suggested a potential role for ILC2-derived IL-13 in collagen accumulation via the downregulation of fibroblast matrix metalloproteinase synthesis in CD patients [73]. However, there are still very limited data available about the role of human ILC2s in gut inflammation.

In the steady state, NCR^+^ ILC3s are the dominant form of ILC3s, comprising 60–75% of total noncytotoxic ILCs, and IL-22 produced by this subset is the major source of this cytokine, which is required for mucosal immunity [14, 74, 75]. Moreover, an initial increase in the production of IL-22 by ILC3s correlates with mucosal healing in human IBD [76]. IL-22, mainly produced by NCR^+^ ILC3s, can be triggered in the steady state by epithelial-adherent commensal microbiota such as adherent-invasive *E. coli* and segmented filamentous bacteria [77, 78]. This homeostatic IL-22 induction has been correlated with mucosal healing in IBD and helps to limit inflammatory colitis [55, 76, 79]. IBD-associated TNF-like ligand 1A (TL1A) production from intestinal mononuclear phagocytes can also induce the release of IL-22 from ILC3s and mediates protection during acute colitis [80].

However, NCR^+^ ILC3s decrease dramatically in chronic inflammation conditions, whereas NCR*^−^* ILC3s are increased in IBD patients [68, 81, 82]. NCR*^−^* ILC3s can contribute to intestinal chronic inflammation [68, 81] through the production of IL-17A and IFN-*γ* [81]. We propose that imbalances in NCR^+^ ILC3s and NCR*^−^* ILC3s lead to disparity in IL-22 and IL-17 production, as a main contributor to the pathology of the IBD (Figure 2). Moreover, chronic colitis might reflect a state of transition from tissue-repairing ILC3s to inflammatory ROR*γ*t^+^ ILC1s [14, 68]. The action of ILC3s in IBD confers is protective in controlling the microbiota, which could inform the development of future therapies that target chronic inflammation via ILC3s.

ILCregs are a regulatory subpopulation of ILCs that can be induced in the intestine and suppress the activation of ILC1s and ILC3s through the release of IL-10, thus playing an inhibitory role in intestinal inflammation [21]. Since the functions of ILCregs have thus far only been studied in mice, their detailed effects are still unclear, necessitating further exploration in humans.

To date, tissue-resident memory ILCs have been investigated in the liver and lungs, but not in the small intestine. Owing to common features such as migration patterns and memory potential, tissue-resident memory ILCs could be regarded as the innate counterparts of T_RM_ cells. T_RM_ cells are a group of self-renewing cells that persist at the site of infection in multiple organs including the intestine and control the development and progression of chronic intestinal inflammation [56]. Therefore, tissue-resident memory ILCs might play a certain role in mediating inflammation of the small intestine, and further research and verification of this are warranted.

## 5. ILCs in Intestinal Cancer

**Figure 3:**
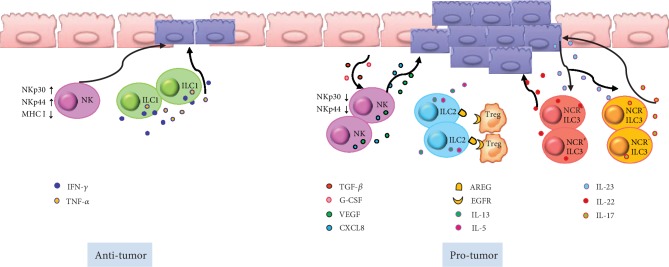
Potential pro- and antitumorigenic roles of ILCs in intestinal cancer. NK cells express high levels of the activating receptors NKp30 and NKp44 and contribute to the fight against tumor cells. Under the influence of transforming growth factor beta 1 (TGF-*β*) and granulocyte colony-stimulating factor (G-CSF), NK cells also have a tumor-promoting effect. ILC1s might be involved in antitumor immunity through the release of IFN-*γ* and TNF-*α*. ILC2-derived cytokines, IL-5 and IL-13, are associated with a high risk of developing inflammation-driven colorectal cancer. ILC2-derived AREG stimulates regulatory T cells and establishes an immune-suppressive tumor microenvironment. In response to IL-23 stimulation, NCR^+^ ILC3s and NCR*^−^* ILC3s mainly produce IL-22 and IL-17, respectively. The IL-23–ILC3–IL-22/IL-17 axis is a critical pathway that promotes tumor growth.

The antitumor function of NK cells in both human and mouse models has been well established [83, 84]. In contrast to tumor-specific cytotoxic T lymphocytes, NK cells, as an important member of the innate immune system, mediate cellular cytotoxicity without priming bispecific antigens [85]. As efficient cytolytic effectors, NK cells can fight against cancer-initiating cells (CICs), which play an important role in malignant tumor recurrence or metastasis [31]. In particular, colorectal cancer-derived CICs are sensitive to NK cell-mediated killing, which is considered related to their high expression levels of ligands that activate NKp30 and NKp44 receptors and low expression levels of major histocompatibility class I molecules that typically inhibit NK cell activity [86] (Figure 3). In different mouse tumor models, the absence of NK cell activation was found to be associated with tumor aggression. Moreover, the absence of a cytotoxic effect of NK cells in colorectal cancer patients before surgery can predict recurrence after local tumor resection [87–89].

However, NK cells might not always act as potent antitumor effectors, as the cytotoxic function of these cells in the tumor microenvironment can be dampened, and NK cells can even have a tumor-promoting effect under certain conditions [85]. The expression of NK cell activation receptors was shown to be decreased in the blood and tumor tissue of colorectal cancer patients [90]. *In vitro*, NK cells cocultured with colorectal cancer cells can release cytotoxic molecules with the impaired production of IFN-*γ* [90]. A possible explanation for the dampened cytotoxicity of NK cells in the tumor microenvironment is the influence of granulocyte colony-stimulating factor (G-CSF) and transforming growth factor beta 1 (TGF-*β*1) [91–94]. In addition, in colorectal cancer patients, tumor-infiltrating NK cells also release high levels of vascular endothelial growth factor and CXCL8 to promote angiogenesis and tumor growth [95] (Figure 3).

Owing to the limited quantity of cytotoxic tumor-infiltrating NK cells and their markedly impaired cytotoxic function in colorectal cancer, the adoptive transfer of activated NK cells has emerged as a potential strategy for clinical therapy. A phase I clinical trial with colorectal cancer patients showed that patients who had previously undergone IgG1-based chemotherapy could tolerate the autologous transfer of NK cells well and could successfully induce type-1 immune responses *in vivo* [96].

Nevertheless, the detailed role of ILC1s in colorectal tumors remains unclear. However, their well-established function in intestinal chronic inflammation could provide a clue based on their potential to create a suitable environment for subsequent malignant transformation [87]. Upon the activation of ILC1, key effector cytokines IFN-*γ* and TNF-*α* are released, and these cytokines promote chronic gut inflammation. IFN-*γ* and TNF-*α* are involved in antitumor immunity [97] (Figure 3). IFN-*γ* plays a role in cell-mediated tumor cell lysis, which inhibits tumor cell proliferation, reduces neoangiogenesis, and suppresses tumor progression [97]. In turn, TNF-*α* can facilitate tumor cell apoptosis and macrophage and dendritic cell infiltration into the local tumor, suggesting direct and indirect antitumor immune responses [98, 99]. However, further research is warranted to illustrate the role of ILC1s in intestinal tumors.

Further, the function of ILC2s during intestinal carcinogenesis has been well researched in both mouse models and humans. Bie's group reported increased numbers of ILC2s, as well as the transcription of ILC2-related genes including *CRTH2*, *GATA3*, and *RORα*, in the peripheral blood of gastric cancer patients [100]. The frequency of ILC2s and the ILC2-related cytokines IL-5, IL-9, and IL-13 were also shown to be increased in patients with UC, which is a condition associated with high colorectal cancer risk due to chronic inflammation [101–103]. ILC2-derived AREG might also stimulate regulatory T cells to establish an immune-suppressive tumor microenvironment [104] (Figure 3). In addition, the ILC2-activated cytokine IL-33 mediates host antitumor immunity, angiogenesis, and stromal remodeling in colorectal cancer pathogenesis [105, 106] and supports the effector functions of cytotoxic NK and CD8^+^ T cells [107]. Thus, further studies should consider the contribution of IL-33 and IL-33-activated ILC2s to the pathogenesis of colorectal cancer.

An increased frequency of ILC3s has also been found in colorectal cancer tissues in a mouse model, and this might play a role in tumor progression [82, 108]. As an ILC3-active factor, the cytokine IL-23 regulates homeostasis and intestinal inflammation. Increased IL-23 expression levels in human colon tumors have been observed, which were associated with tumor progression and worse prognosis [109, 110]. Moreover, IL-23-deficient mice are resistant to tumor formation [109–111], and the depletion of ILCs and IL-22 can reverse established tumors in a Rag1^−/−^ mouse tumor model induced by *Helicobacter hepaticus* oral infection, indicating that ILCs and IL-22 are essential for the formation of colonic tumors. ILC3s are the main producers of IL-22, which generally has a tumor-promoting effect, but its condition-dependent nature has been proposed with different roles observed in different cancer microenvironments [112–114]. As another important cytokine produced by ILC3s, IL-17A has been implicated in human colorectal cancer, and increased IL-17-producing cells could independently predict worse clinical outcomes [115, 116] (Figure 3). A recent study showed that IL-23 can induce the conversion of ILC1s to ILC3s, demonstrating that the IL-23–ILC3–IL-17 axis is a critical pathway that promotes tumor growth [117]. Collectively, these results showed that imbalances in ILC3s could contribute to the progression of colorectal cancer and that IL-17 and IL-22 could be potential treatment targets.

Finally, monobenzone-induced memory CD49b^+^ cNK cells were found to effectively control B16 tumor development in a mouse model [118]. Although there has been no study of the function of tissue-resident memory ILCs in intestinal cancer, as the apparent innate counterparts of T_RM_ cells, they are considered to have potential benefits in long-term tumor control and vaccination, with promising clinical value for tumor immunotherapies and vaccine-development strategies [23].

## 6. Concluding Remarks

Mounting evidence has now demonstrated that ILCs are critical regulators of intestinal homeostasis, inflammation, and cancer. Although tremendous progress has been made in understanding the detailed roles of ILCs in the restoration of epithelial barrier integrity and protection against infiltrating pathogens, and intestinal inflammation and cancer, this research has also revealed that ILCs represent a highly heterogeneous group of cells. The different groups and subsets of ILCs and their corresponding cytokines have now emerged as important mediators of various pathological conditions, and even the same subtype might have diverse roles in different contexts or at different stages of disease. Moreover, ILCs also play roles in metabolic homeostasis and contribute to the pathogenesis of graft-versus-host disease. Thus, further studies focused on exploring the regulation and pathophysiology of ILCs might reveal potential targets for future therapeutic interventions.

## Figures and Tables

**Table 1:** Characteristics of intestinal innate lymphoid cells (ILCs).

	NKs	ILC1s	ILC2s	ILC3s	ILCregs
Transcription factors	T-bet	T-bet	GATA3, ROR*α*	ROR*γ*t	Id3
Active factors	TGF-*β*, GM-CSF	IL-15	IL-25, IL-33, TSLP	IL-23	TGF-*β*
Effective factors	IFN-*γ*, IL-22, VEGF, CXCL8	IFN-*γ*, TNF-*α*	IL-5, IL-13	IL-22, IL-17, GM-CSF, IFN-*γ*	IL-10
